# Correlations between chromaticity parameters and shear strength of granite residual soil with different free iron oxide content and moisture content

**DOI:** 10.1038/s41598-026-38135-0

**Published:** 2026-02-01

**Authors:** Zhibing Wang, Wei Deng, Changwei Diao, Xiao Kuang, Jian Yin

**Affiliations:** 1 Guangxi Key Laboratory of Geomechanics and Geotechnical Engineering, College of Civil Engineering, Guilin University of Technology, No.12, Jiangan Road, Guilin, 541004 China; 2 School of Civil Engineering, Guilin University of Technology, Guilin, 541004 China

**Keywords:** Granite residual soil, CIE-l*a*b* color model, Free iron oxide content, Water content, Shear strength, Engineering, Environmental sciences, Materials science

## Abstract

In hot-humid regions, granite residual soil’s moisture and free iron oxide contents show marked spatiotemporal heterogeneity tied to seasons and weathering, creating an inherent connection between its visual color and mechanical properties. This study carried out indoor chromaticity parameter measurements and shear strength tests on residual granite soils in southeastern Guangxi with varying moisture and free iron oxide contents, acquiring CIE-L*a*b* color indices and shear strength parameters. Using path and regression analyses, it explored correlations between chromaticity parameters (L* [lightness], a* [red-green chroma], b* [yellow-blue chroma]) and shear strength indices (internal friction angle* φ*, cohesion *c*). Results showed that at constant moisture, rising free iron oxide content positively correlates with cohesion, a* and b*, but not with L*; *φ* has no significant links to any chromaticity parameter. At fixed free iron oxide content, cohesion has a nonlinear correlation with L*, a* and b* as moisture increases, while *φ* is significantly positively correlated with these chromaticity indices. The findings verify that chromaticity parameters can effectively and rapidly estimate granite residual soil’s shear strength, providing valuable references for geotechnical evaluations.

## Introduction

Granite residual soil, a distinctive geotechnical material formed through the weathering of granite under hot and humid climates, is widely distributed in the mountainous regions of southern China. This soil type is characterized by unique physical and mechanical properties, including a high void ratio^1^, low permeability^2^, and a pronounced tendency to soften and disintegrate upon wetting^3^. These characteristics are intrinsically linked to prevalent local geohazards, such as landslides and soil erosion (e.g., collapsible gullies), and pose significant challenges in geotechnical engineering, leading to risks like slope and foundation pits instability^4,5^. Rapid determination of soil strength parameters is essential for slope stability assessment^6^. Fast and cost-effective alternative methods for estimating soil physical and mechanical characteristics using color parameters remain one of the main techniques used by most soil scientist onto the soil properties assessment^7–9^.

Shear strength and color, two typical macroscopic properties of soil, are governed by its internal material composition, microstructure, and external environmental conditions^10–12^. The shear strength of soil, a critical parameter for stability assessment, is governed by multiple factors. Extensive research has identified key influences, such as water content^13,14^, dry density^15,16^, particle gradation^17^, and material composition^18,19^. Among these, soil color serves as an optical indicator of its intrinsic properties, reflecting variations in mineralogy, organic matter, water content, and microstructure. Spectral parameters derived from color spaces (e.g., RGB, CIE-L*a*b*) or hyperspectral data can effectively capture these subtle compositional variations^20,21^. Notably, water content (*ω*) and free iron oxide (*Fe*_*d*_) content are two primary factors that not only induce changes in soil color but also fundamentally control shear strength. For instance, while an optimal water content can enhance cohesion and the internal friction angle (*φ*), excessive water lubricates particles, thereby reducing shear strength^22,23^. This interplay is often visually manifested, as increasing water content typically darkens the soil^24^.

The primary constituents of granite residual soil consist primarily of minerals (e.g., kaolinite and quartz), along with trace to minor amounts of iron oxides and their hydrates. Although iron oxides occur at low contents with heterogeneous distribution in the soil, they remain key factors that profoundly influence soil structure and its physical and mechanical properties, free iron oxide forms cement films or bridging structures on the surface of soil particles, and enhances particle bonding through chemical bonding and interparticle attraction, thereby increasing soil cohesion^25^. The most prevalent mineral forms of free iron oxide in granite residual soil are hematite and goethite, which serve as the primary sources of the soil’s red and yellow–brown hues^26,27^.

As key factors cooperatively regulating the color and shear strength of granite residual soil, water content and free iron oxide content exhibit spatiotemporal variability driven by ambient environmental fluctuations, and they serve as the intrinsic drivers underpinning variations in soil properties. The hot-humid climate induces frequent fluctuations in the water content of granite residual soil between 5 and 34%^28^; meanwhile, its free iron oxide content is also characterized by dynamic variations and spatial heterogeneity^29,30^. The surface color and shear strength of granite residual soil thus display pronounced spatiotemporal variability. Developing methods to characterize the material properties of granite residual soil and estimate its shear strength based on surface color variations holds considerable implications for slope stability assessment, thereby warranting further in-depth investigation.

Therefore, this study focuses on granite residual soil from southeastern Guangxi as the research subject. Through laboratory experiments, it quantifies the variation patterns of the soil’s CIE-Lab chromaticity parameters (i.e., L*, a*, b*) and shear strength indices (i.e., internal friction angle *φ*, cohesion c) under varying water contents and free iron oxide contents. Employing statistical analysis, it systematically explores the intrinsic correlation mechanisms between chromaticity parameters and shear strength, furthermore, it establishes estimation models linking easily measurable chromaticity parameters to soil shear strength indices, providing a valuable reference for the property estimate of granite residual soil and slope stability assessment.

## Materials and methods

### Test soil samples

**Fig. 1:**
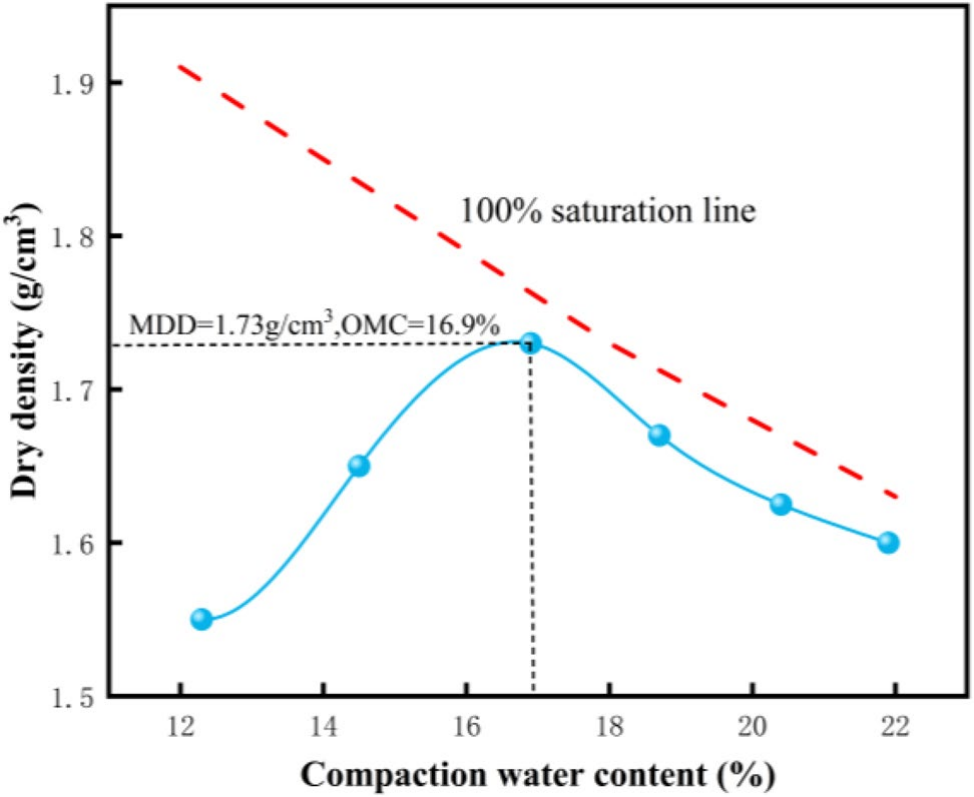
Standard proctor compaction curve of sampled soil.

Test specimens of granite residual soil were collected from Guquan Village, Rong County, Yulin City, Guangxi Zhuang Autonomous Region—a site where the soil is representative of regional granite residual soil characteristics. The specimens appear reddish-brown, with a notably high proportion of silt and sand fractions. Grain-size analysis indicated the particle-size distribution: 40.98% sand, 32.28% silt, and 26.64% clay, resulting in a combined silt–clay content of 58.92% (surpassing 50%). Atterberg limits tests yielded a liquid limit (LL) of 45.8%, a plastic limit (PL) of 30.3%, and thus a plasticity index (PI) of 15.5. Plotting these indices on the Unified Soil Classification System (USCS) Plasticity Chart places the soil within the Lean Clay (CL) category; given its substantial sand content, it is further classified as Clay with Sand (CL) per USCS criteria. To assess the compaction behavior of this Clay with Sand (CL) soil, a Standard Proctor compaction test was conducted, with the corresponding compaction curve depicted in Fig. 1. Key physical properties of the soil are summarized in Table 1.

**Table 1:** Properties of sampled soil.

Property	Value
Natural water content/%	26.2
Specific gravity	2.71
Natural density/(g/cm^3^)	1.50
Liquid limit	45.8
Plasticity limit	30.3
Plasticity index	15.5
Grain-size distribution (%)	
Sand (0.075–2 mm)	40.98
Silt (0.005–0.075 mm)	32.28
Clay (< 0.005 mm)	26.64
Mineralogy (%)	
Quartz	45–55
Kaolinite	20–30
Illite	5–10
Feldspar	< 5%
Free iron oxides FIO (g/kg)	23.508

### Sample preparation

**Fig. 2:**
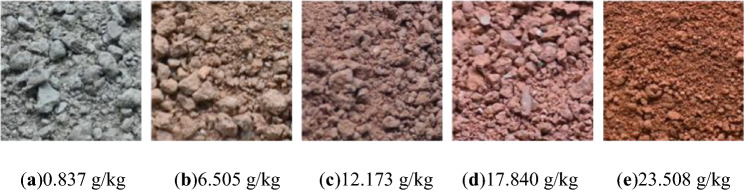
Appearance of granite residual soil samples with different free iron oxide contents.

The preparation process of soil samples with different free iron oxide contents is as follows: Firstly, the reduction-complexation method (DCB method) was used to remove most of the free iron oxide from the soil. Then, the iron-removed soil sample was mixed with the original soil sample in a certain proportion to obtain the test soil samples, the resulting soil samples were subjected to 5 wet-dry cycles to form new effective connections between aggregates in the soil, each wet-dry cycle consists of two steps: dehumidification and humidification. For dehumidification, the soil samples were dried in a constant-temperature oven at 40 °C for 42 h; for humidification, a vacuum saturation device was used to saturate the soil with water, and the saturated water content was 32.4%. Finally, a visible spectrophotometer was used to accurately determine the free iron oxide content of the prepared soil samples. The actual free iron oxide contents of the soil samples were 0.837, 6.505, 12.173, 17.840, and 23.508 g/kg, the corresponding photographs are shown in Fig. 2. respectively, the prepared soil samples with different free iron oxide contents are shown in the air-dried granite residual soil samples with the above-mentioned different free iron oxide contents were used, and the amount of water to be added was calculated according to the water content gradients of 5%, 10%, 15%, 20%, and 25%. The water was sprayed evenly, and the samples were sealed and allowed to stand for 24 h, a small part of the soil sample was then placed in an aluminum box and dried at 105 °C to a constant weight to calculate the actual water content. If the error between the actual water content and the configured value was less than 1%, the test was continued, otherwise, the samples were re-prepared.

### Test methods

#### Soil color measurement test

**Fig. 3:**
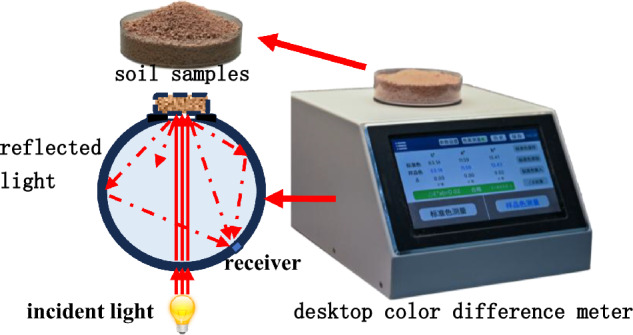
Principle of the desktop color difference meter.

Soil sample color was measured using the CIE-L*a*b* color space—a widely recognized perceptually uniform color system. In this system, each color is defined by three scalar chromaticity parameters (Cartesian coordinates): L* [lightness], ranging from 0 (absolute black) to 100 (absolute white); a* [red-green chroma], where positive values indicate red and negative values indicate green; and b* [yellow-blue chroma], where positive values indicate yellow and negative values indicate blue. In this study, a high-precision benchtop colorimeter (Model LS177) was employed to quantify the chromaticity parameters of test samples (Fig. 3). Equipped with a 50-mm measuring aperture, it is suitable for analyzing samples with rough, uneven surfaces (e.g., soil). Test soil samples with varying water contents (*ω*) and free iron oxide contents (*Fe*_*d*_) were placed in the colorimeter’s measuring tray, and their L*, a*, and b* values were recorded. Each sample was measured at least three times, with the average value adopted as the final chromaticity parameter.

#### Direct shear test

Soil shear strength was quantified using a Model ZJ strain-controlled direct shear apparatus, which enables precise regulation of shear displacement and rate for soil specimens, thereby ensuring accurate determination of shear strength indices under loading. Test specimens with varying free iron oxide contents and moisture contents were prepared into cylindrical samples (61.8 mm in diameter, 20 mm in height) via the standard static compaction method. The dry density of all specimens was uniformly controlled at 1.50 g/cm^3^ to ensure test consistency. Post-preparation, each specimen was cured in a humidity-controlled chamber for 24 h for moisture equilibration prior to testing. Applied normal stresses were 100, 200, 300, and 400 kPa, with a constant shear rate of 0.8 mm/min (a standard quick shear rate to restrict pore water dissipation). Loading was terminated when the shear displacement reached 6 mm. Following the tests, the experimental data were analyzed to derive the soil’s shear strength indices—internal friction angle (*φ*) and cohesion (*c*)—under consolidated quick shear conditions.

#### Establishment of shear strength estimate model based on chromaticity parameters

To establish correlations between chromaticity parameters (L* [lightness], a* [red-green chroma], b* [yellow-blue chroma]) and shear strength indices (internal friction angle *φ*, cohesion *c*), and enable rapid inference of shear strength from chromaticity parameters, path analysis was employed to develop a correlation model. The approach proceeded as follows: First, examine the intrinsic associations between chromaticity parameters and soil water content (*ω*) and free iron oxide content (*Fe*_*d*_);Second, elucidate the influencing mechanisms of ω and Fed on shear strength indices; Finally, quantify the relationships between chromaticity parameters and shear strength indices by accounting for the mediating roles of intermediate variables (i.e., *ω* and *Fe*_*d*_).

#### Validation of the shear strength estimate model

Following the establishment of the chromaticity-based shear strength prediction model, 20 independent test datasets were utilized to validate its predictive performance. These datasets were obtained from additional granite residual soil samples collected from adjacent townships of Rong County, Yulin City, Guangxi Zhuang Autonomous Region—ensuring geographic proximity to the original sampling site (Guquan Village) while maintaining data independence. The test protocols for these independent samples were strictly consistent with those of the modeling dataset: undrained direct shear tests were conducted on specimens prepared via static compaction at a controlled dry density of 1.50 g/cm^3^, eliminating experimental variability and ensuring comparability between datasets. The chromaticity parameters (L*, a*, b*) of the independent samples were then input into the proposed model to generate predicted values of shear strength indices (internal friction angle *φ* and cohesion *c*), which were subsequently compared with their corresponding laboratory-measured values.

## Results

### Soil color measurement test results

**Fig. 4:**
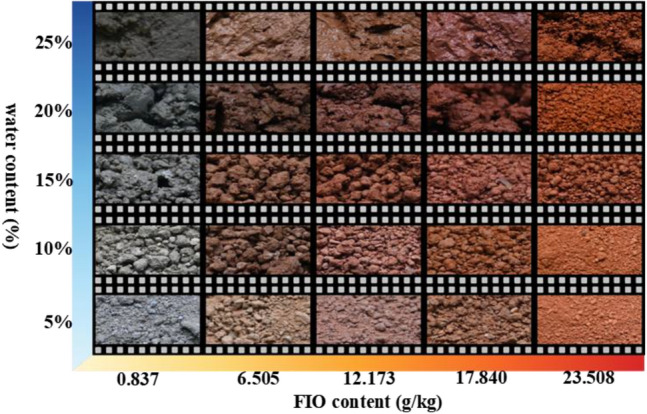
Photographs of granite residual soil under different water contents and FIO contents.

Surface colors of granite residual soil under varying water contents (*ω*) and free iron oxide contents (*Fe*_*d*_) are illustrated in Fig. 4. As illustrated in figure, changes in *ω* and *Fe*_*d*_ exert a highly significant influence on soil color. Overall, as water content increases, soil color gradually darkens; as *Fe*_*d*_ increases, soil color gradually reddens.

**Fig. 5:**
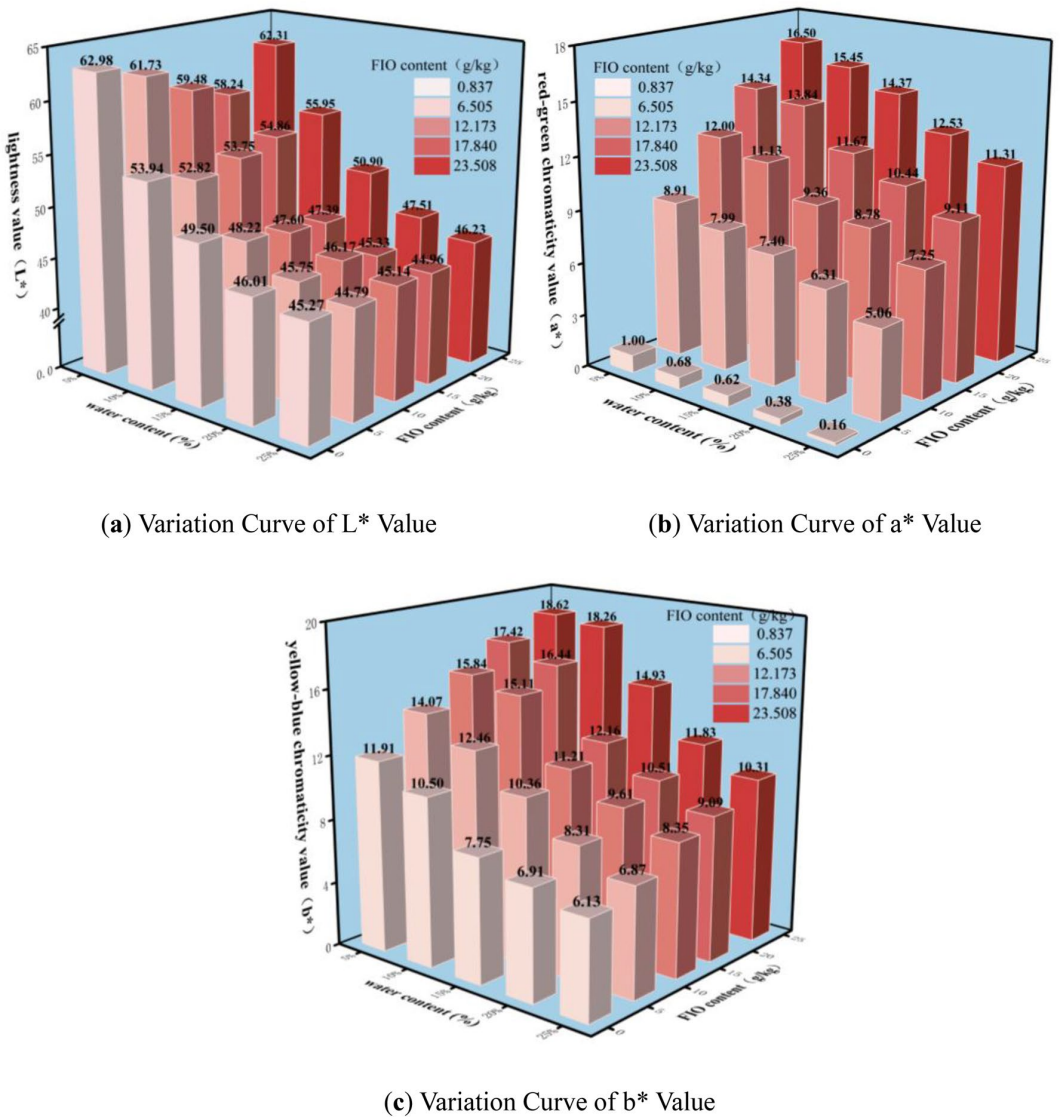
Bar chart of variations in lab values of granite residual soil under different water contents and FIO contents.

Chromaticity parameters (L* [lightness], a* [red-green chroma], b* [yellow-blue chroma]) of granite residual soil under varying water contents (*ω*) and free iron oxide contents (*Fe*_*d*_) were quantified via a colorimeter, with results illustrated in Fig. 5. As shown in Fig. 5a, L* is strongly influenced by *ω*: L* values for all *Fe*_*d*_ levels decrease with increasing *ω*. However, at a constant *ω*, *Fe*_*d*_ exerts no significant effect on L*. For example, at *Fe*_*d*_ = 23.508 g/kg: as *ω* increased from 5 to 10%, L* decreased from 62.31 to 55.95 (a 10.2% reduction); when *ω* further increased to 15%, L* dropped to 50.90 (a 9.03% decrease); as *ω* rose from 20 to 25%, the decrease was merely 2.7%. These results confirm that L* is primarily regulated by *ω*, decreasing with increasing *ω*. In contrast, *Fe*_*d*_ has no significant impact on L*, indicating that inherent color variations of granite residual soil exert minimal influence on the lightness parameter.

As illustrated in Fig. 5b,c, a* and b* exhibit a broadly consistent response to *ω* and *Fe*_*d*_. At a constant *ω*, both a* and b* increase with increasing *Fe*_*d*_ (exhibiting a positive correlation). At a constant *Fe*_*d*_, a* and b* decrease with increasing *ω* (exhibiting a negative correlation). Notably, the variation range of b* is slightly wider than that of a* with changes in *ω*, whereas a*’s variation range is substantially wider than that of b* with changes in *Fe*_*d*_. For instance, at *Fe*_*d*_ = 23.508 g/kg: as *ω* increased from 0 to 25%, a* decreased from 16.5 to 11.31 (a 31.5% reduction), while b* decreased from 18.62 to 10.31 (a 51.3% reduction)—demonstrating that b* exhibits a wider variation range than a* with *ω* changes. At *ω* = 5%: as *Fe*_*d*_ increased from 0 to 23.508 g/kg, a* rose from 1 to 16.5, whereas b* increased by merely 6.71. These results further confirm that a* is more sensitive to variations in *Fe*_*d*_.

### Direct shear test results

**Fig. 6:**
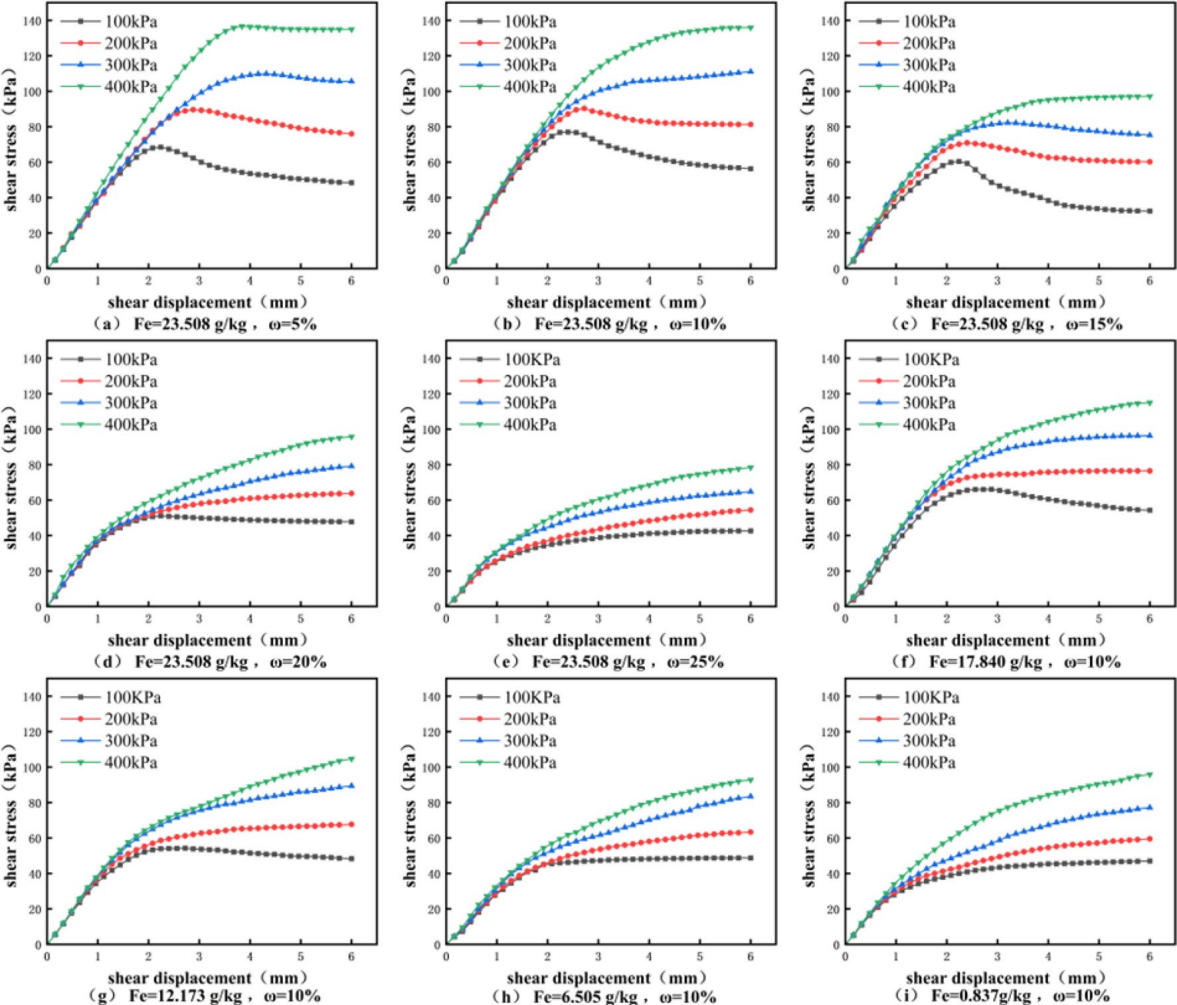
Stress–strain curves of granite residual soil under different water contents and free iron oxide contents.

As illustrated in Fig. 6, shear stress–shear displacement curves of granite residual soil under varying water contents (*ω*) and free iron oxide contents (*Fe*_*d*_) exhibit distinct characteristics. Notably, the peak values of these curves display a consistent decreasing trend with decreasing *Fe*_*d*_ and increasing *ω*. Based on the presence of stress peaks, the curves can be broadly categorized into two types: Case 1: *ω* < 15% and *Fe*_*d*_ > 50% – The stress–strain curves of the residual soil exhibit distinct stress peaks, classified as strain-softening behavior. During loading, shear stress first undergoes linear elastic growth with increasing axial strain, after reaching the stress peak, it either stabilizes or decreases gradually before attaining a steady state. Case 2: *ω* > 15% and *Fe*_*d*_ < 50% – The stress–strain curves gradually transition from strain-softening to strain-hardening behavior. Specifically, the curve shows linear growth in the early loading stage, followed by a gradual diminishment of the slope, and finally shear stress stabilizes or exhibits a slight increase. These results indicate that increasing *ω* and decreasing *Fe*_*d*_ significantly impair the soil’s structural integrity, leading to the absence of strain-softening during shearing.

**Fig. 7:**
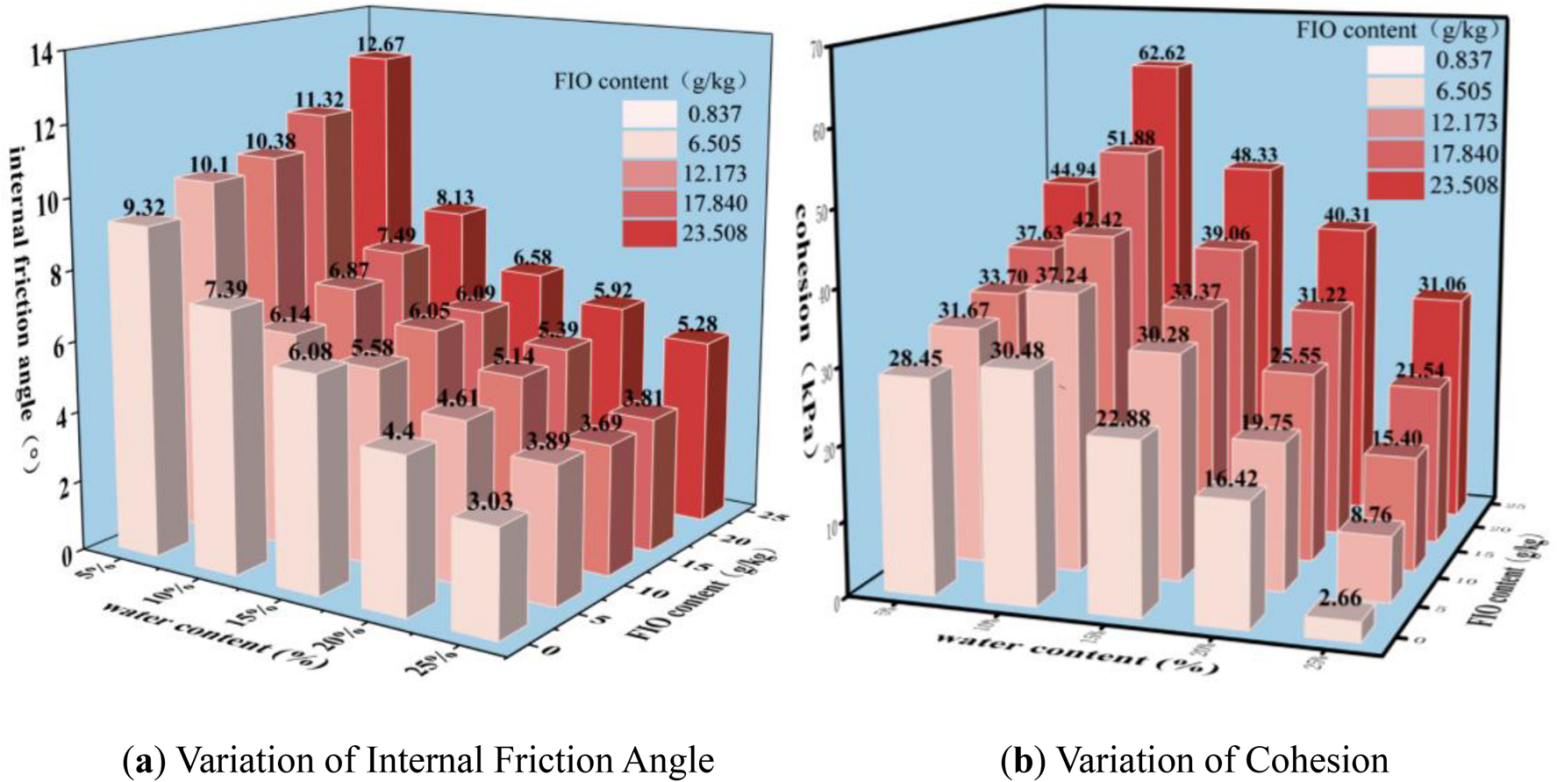
Variations of cohesion (*c*) and internal friction angle (*φ*) of Granite residual soil under different water contents and free iron oxide contents.

Failure shear strengths of granite residual soil under varying conditions were determined via direct shear tests. Based on this, consolidated quick shear shear strength indices (internal friction angle *φ*, cohesion *c*) were derived using the Mohr–Coulomb criterion (Fig. 7). As illustrated in Fig. 7a, *φ* decreases with increasing *ω*, and the rate of *φ* reduction gradually diminishes as *ω* rises from 5 to 25%. This phenomenon indicates that water acts as a lubricant between soil particles, with this effect being particularly pronounced at low *ω*. In contrast, *φ* exhibits no significant variation with increasing *Fe*_*d*_, demonstrating that *Fe*_*d*_ exerts minimal influence on the internal friction angle of granite residual soil. For example, at *Fe*_*d*_ = 23.508 g/kg: as *ω* increased from 5 to 10%, *φ* decreased from 12.67 to 8.13 (a 35.8% reduction); between *ω* = 10% and 15%, *φ* decreased by 19.1%; at *ω* = 20%, the reduction rate dropped to merely 10%.

In contrast, cohesion (*c*) exhibits distinct behavior (Fig. 7b): *c* first increases and then decreases with increasing *ω*, while it rises consistently with increasing *Fe*_*d*_ (exhibiting a strong positive correlation). When *ω* increased from 5 to 10%: for *Fe*_*d*_ = 0.837 g/kg, c varied by only 7.1%; for *Fe*_*d*_ = 23.508 g/kg, the variation reached 39.4%—nearly six times that of the former.

This observation confirms that *Fe*_*d*_ is critical to the cohesion of granite residual soil, with water acting as a solvent in this process: water permeates soil pores and reacts with chemical components (e.g., *Fe*_*d*_) to form cementitious substances, which fill interparticle gaps and enhance cohesion, however, insufficient water limits cementitious formation, while excessive water introduces free water that disrupts cementation^31^, consequently, *c* of granite residual soil first increases and then decreases with increasing *ω*^32^.

### Shear strength estimate model

#### Chromaticity parameters and internal friction angle

**Fig. 8:**
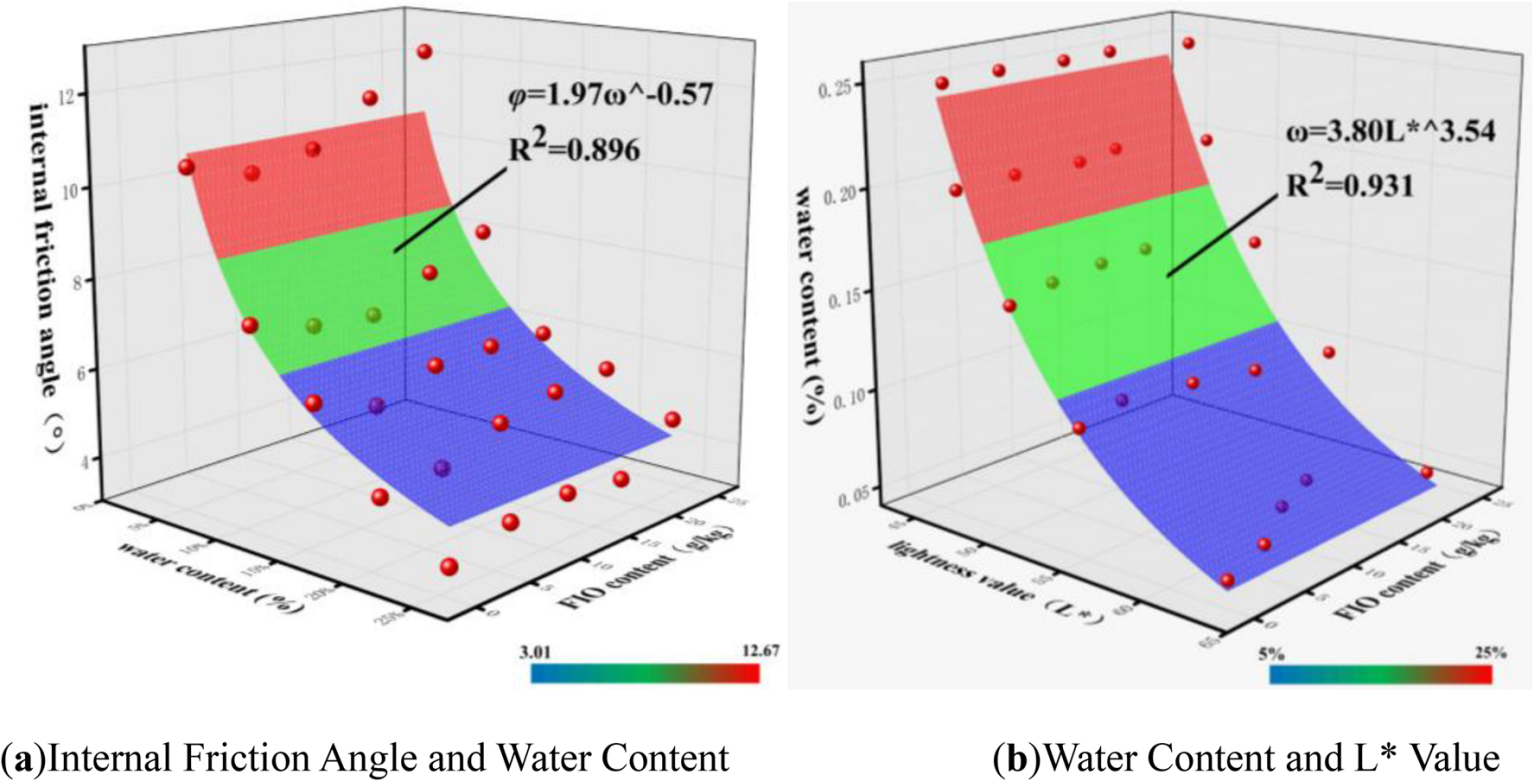
Surface fittings between internal friction angle and water content, and between water content and L value of granite residual soil.

As can be seen from Fig. 7a, for each free iron oxide content (*Fe*_*d*_), the changes in internal friction angle (*φ*) caused by variations in water content (*ω*) all exhibit a non-linear attenuation trend, which conforms to a first-order exponential curve:

1 $$\varphi = A_{1} \omega^{{B_{1} }}$$

where *φ* denotes the internal friction angle, *ω* denotes the water content, and A_1_ and B_1_are both coefficients. Existing data were fitted using the formula, and the fitting results are shown in Fig. 8a.

As can be seen from Fig. 5a, the water content (*ω*) exhibits a strong correlation with the lightness value (L*), and the water content (*ω*) of granite residual soil can be expressed by the chromaticity parameter (L*):

2 $$\omega = A_{2} L*^{{B_{2} }}$$

where *ω* denotes the water content, L*denotes the lightness value, and A_2_ and B_2_ are both coefficients. Existing data were fitted using the formula, and the fitting results are shown in Fig. 8b.

Based on Eqs. (1) and (2), we can derive the expression for the internal friction angle (*φ*) in terms of the lightness value (L*):

3 $$\varphi = AL*^{B}$$

**Fig. 9:**
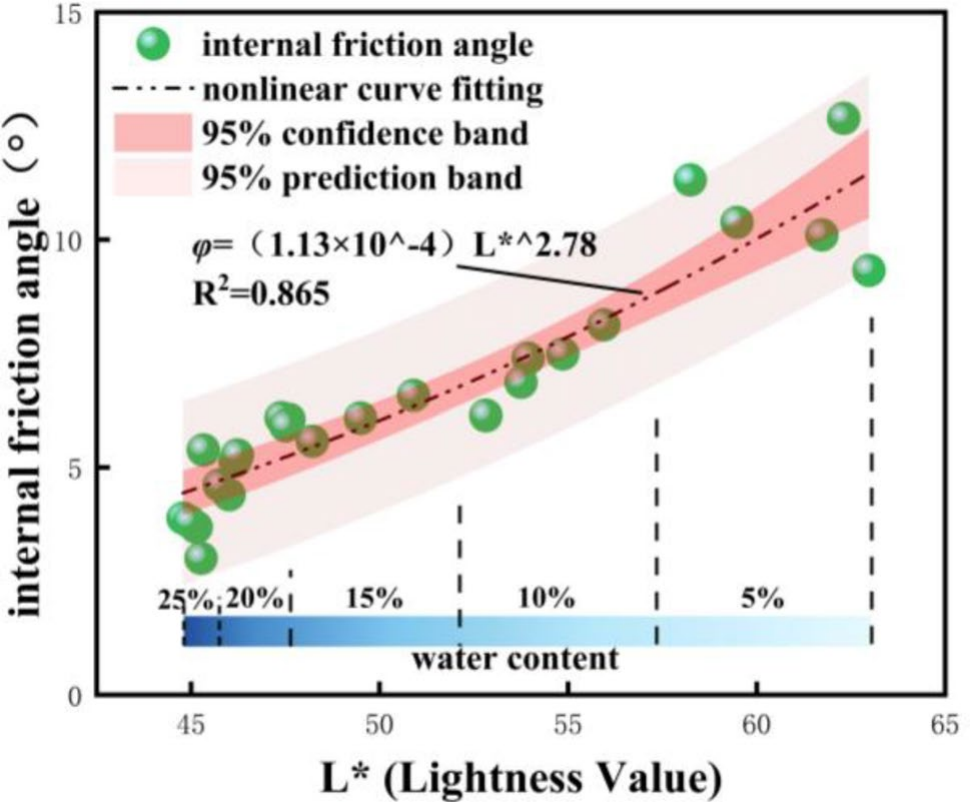
Fitted curve between internal friction angle and L value.

Linear fitting (Eq. (3)) was conducted to correlate lightness parameter L* with internal friction angle (*φ*), with results illustrated in Fig. 9. As shown in the figure, *φ* exhibits an increasingly wide variation range with increasing L*, meanwhile, sample points cluster more tightly at low L* values. This phenomenon can be explained as follows: a colorimeter quantifies chromaticity parameters by detecting reflected light intensity from the sample surface under a fixed light source. Increasing water content (ω) replaces diffuse reflection at the air-particle interface on the soil surface with specular reflection at the water-particle interface, thereby reducing overall reflected light intensity. Notably, this reduction is more pronounced at low ω.

#### Chromaticity parameters and cohesion

As illustrated in Fig. 7b, cohesion (*c*) of granite residual soil is jointly regulated by water content (*ω*) and free iron oxide content (*Fe*_*d*_); thus, the combined effects of these two factors are analyzed herein. Since red-green chroma (a*) exhibits a strong correlation with *Fe*_*d*_—and as shown in Fig. 5b—at a constant ω, *Fe*_*d*_ can be characterized by the chromaticity parameter a*:

4 $$Fe_{d} = C_{1} a*^{{D_{1} }}$$

where *Fe*_*d*_ denotes the free iron oxide content, a* denotes the red-green chromaticity value, and C_1_ and D_1_ are both coefficients.

As illustrated in Fig. 5b, a* exhibits a linear decrease with increasing *ω*, thus, by introducing the independent variable *ω*, the expression for *Fe*_*d*_ under varying *ω* is derived:

5 $$Fe_{d} = C_{2} a*^{{D_{2} }} + E\omega$$

where *ω* denotes the water content, and C_2_, D_2_, and E are all coefficients.

**Fig. 10:**
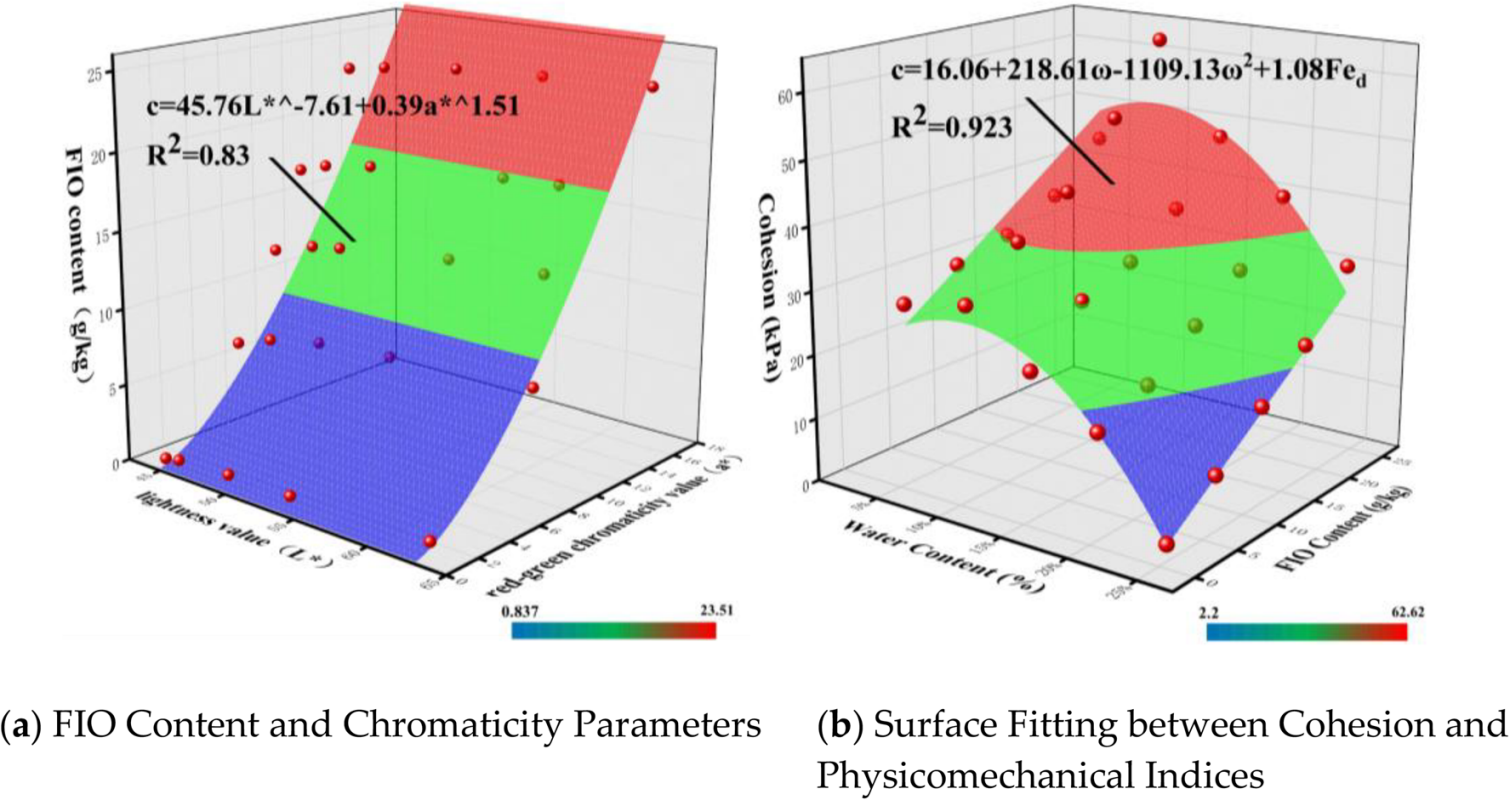
Surface fittings between FIO content and chromaticity parameters (L* and a*), and between cohesion and physicomechanical indices (ω and FIO content) of granite residual soil.

Among them, according to Eq. (2), the water content (*ω*) can be expressed by the lightness value L*, so the formula can be rewritten as:

6 $$Fe_{d} = A_{3} L*^{{B_{3} }} + C_{3} a*^{{D_{3} }}$$

where L* denotes the lightness value, and A_3_, B_3_, C_3_ and D_3_ are all coefficients. The data were fitted using the formula, and the fitting results are shown in Fig. 10a.

As can be seen from Fig. 7b, under the condition of constant free iron oxide content (*Fe*_*d*_), the variation curve between cohesion (*c*) and water content (*ω*) conforms to the relationship of a quadratic function:

7 $$c = F_{1} + G_{1} \omega + H_{1} \omega^{2}$$

where *c* denotes the cohesion, ω denotes the water content, and F_1_,G_1_, andH_1_ are all coefficients.

Under the condition of constant water content (*ω*), the cohesion (*c*) of granite residual soil increases with the increase of free iron oxide content (*Fe*_*d*_). Therefore, on the basis of Eq. (7), the following formula can be obtained by introducing the variable of free iron oxide content (*Fe*_*d*_):

8 $$c = F_{2} + G_{2} \omega + H_{2} \omega^{2} + IFe_{d}$$

where *Fe*_*d*_ denotes the free iron oxide content, and F_2_,G_2_,H_2_, and I are all coefficients. The data were fitted using Eq. (8), and the fitting results are shown in Fig. 10b.

By substituting Eqs. (1) and (6) into Eq. (8) and simplifying them, the conversion formula between the chromaticity parameters of granite residual soil and its cohesion can be obtained:

9 $$c = C + DL + EL^{2} + Fa^{G}$$

**Fig. 11:**
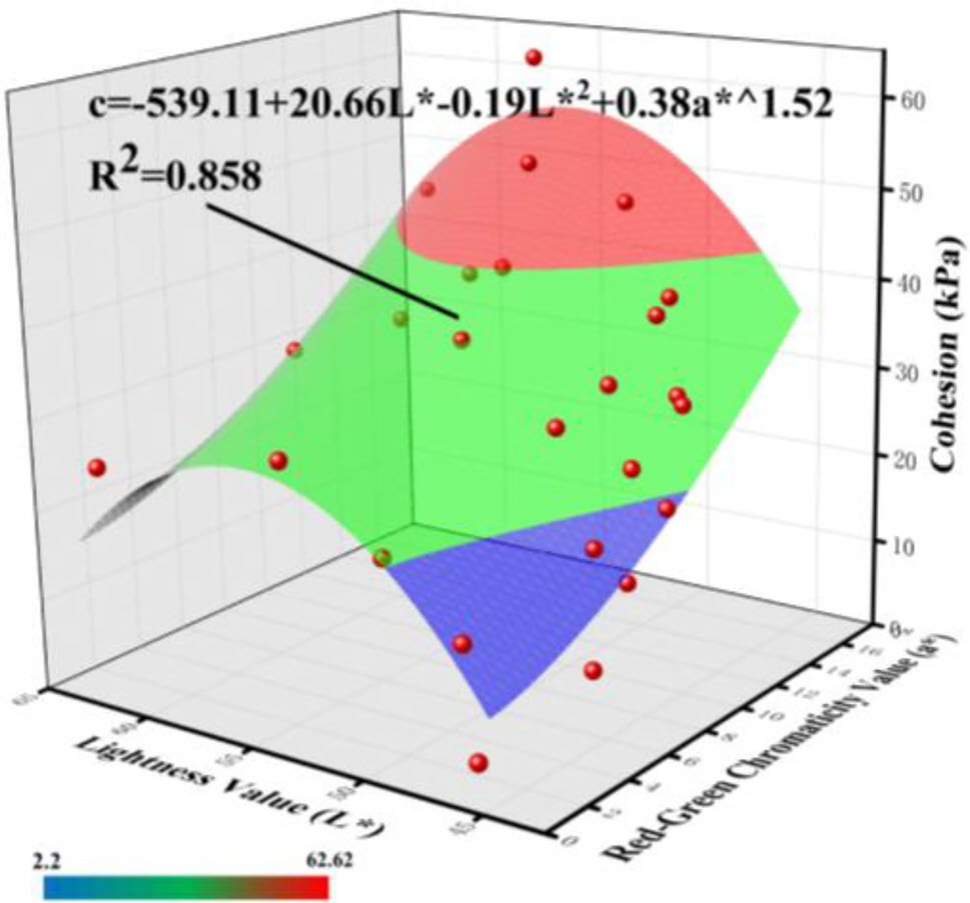
Fitted curve between cohesion and chromaticity parameters (L* and a*).

Fitting was performed between the lightness value (L*), red-green chromaticity value (a*), and internal friction angle (*φ*) using Eq. (9), and the fitting results are shown in Fig. 11.

### Validation of the shear strength estimate model

**Fig. 12:**
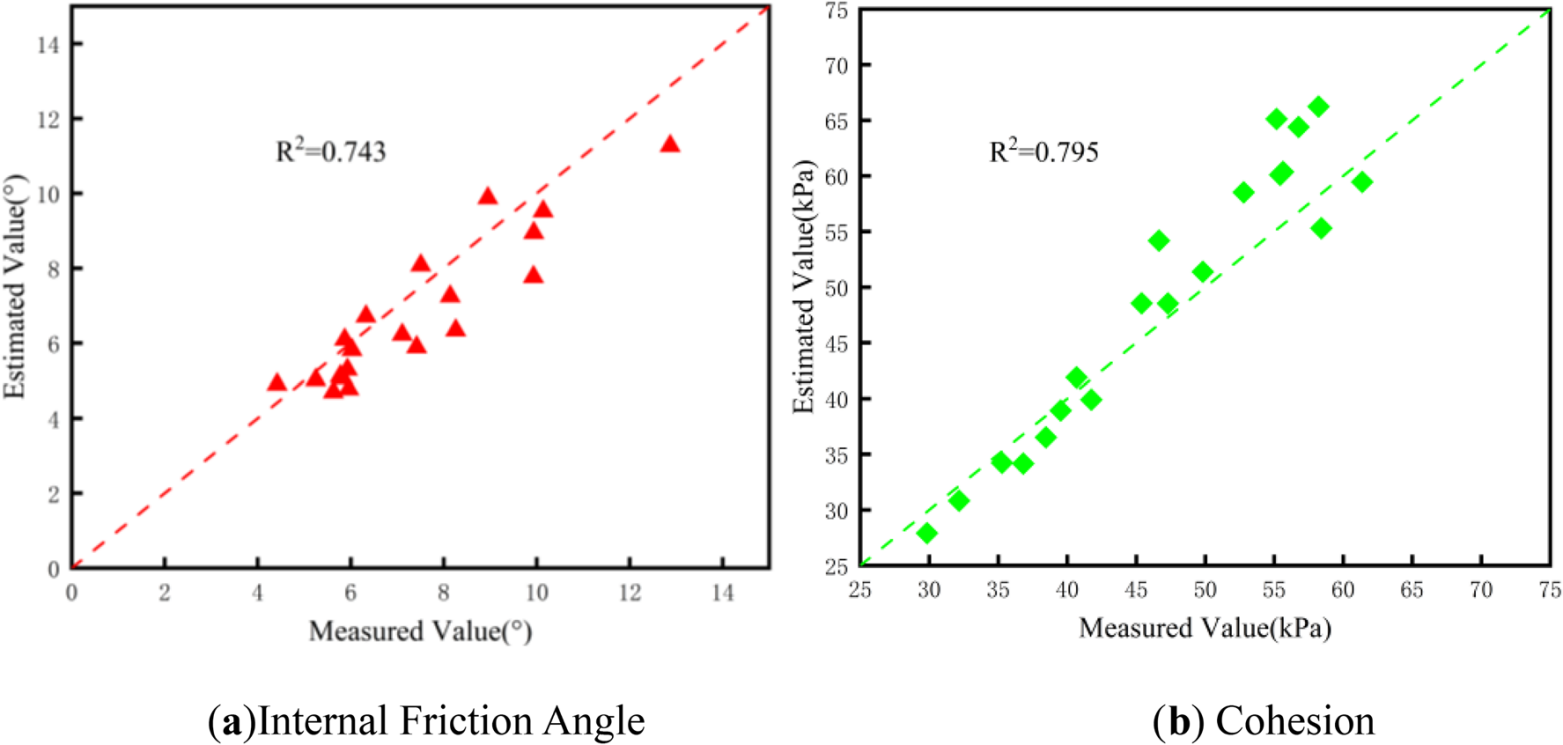
Scatter plot of measured vs. estimated shear strength indices for granite residual soil with y = x fitting line.

To verify the model’s accuracy, 20 independent granite residual soil samples (collected from adjacent townships of Rong County, Yulin) were used—all prepared/tested with the same protocol as the modeling dataset (static compaction, consolidated quick shear tests). Chromaticity parameters (L*, a*, b*) of these samples were input into the model to get estimated shear strength values, then compared with measured values (Fig. 12). Figure 12 shows the measured vs. estimated scatter plots (with y = x as ideal consistency reference): For internal friction angle (*φ* Fig. 12a), R^2^ = 0.743 (the model explains 74.3% of *φ* variation, data points cluster near the fitting line). For cohesion (*c* Fig. 12b), R^2^ = 0.795 (79.5% of *c* variation is explained, with tighter data distribution).These results confirm the model’s reliability: it can rapidly estimate granite residual soil’s shear strength in Yulin via simple chromaticity measurements.

## Discussion

### Effects of different water contents and free iron oxide contents on the shear strength parameters of granite residual soil

This study confirms that water content (*ω*) and free iron oxide content (*Fe*_*d*_) exert a significant influence on the shear strength indices of granite residual soil, consistent with previous studies^33,34^.

Soil cohesion (*c*) first increases and then decreases with increasing *ω*, this is attributed to the fact that *c* is primarily associated with interparticle gravitational forces and cementation strength^35^. At low *ω*, electrostatic attraction between soil particles is relatively strong, an optimal ω facilitates tight, effective cementation between particles. As *ω* continues to increase, water fills interparticle voids and forms a water film on particle surfaces, reducing interparticle gravitational forces and thus lowering *c*^36^. Cementation strength, by contrast, is governed by *Fe*_*d*_: lower *Fe*_*d*_ corresponds to weaker cementation and, consequently, lower *c*^25^.

Internal friction angle (*φ*) is primarily influenced by *ω*, with two key mechanisms: For one thing, at low *ω*, soil particles are primarily in direct contact, with their rough surfaces creating mechanical interlocking and thus high frictional resistance. As *ω* increases, the adsorbed water film between particles thickens, forming a lubricating layer that reduces interparticle sliding resistance and consequently lowers *φ*^37^. For another, increased *ω* induces the dissolution of cementing materials (e.g., carbonates, iron-manganese oxides) in the soil, resulting in the loss of interparticle cementation and a subsequent reduction in *φ*. Furthermore, hydrophilic clay minerals (e.g., illite) in the soil absorb water and swell, increasing interparticle repulsive forces, loosening the soil structure, and further lowering *φ*^38^.

### Effects of different water contents and free iron oxide contents on the chromaticity parameters of granite residual soil

**Fig. 13:**
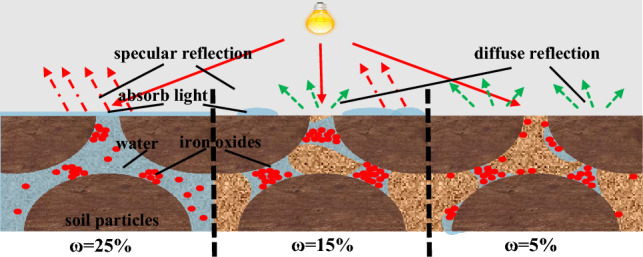
Mechanism analysis diagram of water content and free iron oxide content influencing color parameters of granite residual soil.

In granite residual soil, free iron oxide content (*Fe*_*d*_) and water content (*ω*) directly affect its shear strength indices, while soil color changes effectively capture these variations. As illustrated in Fig. 13.

L* (lightness), a* (red-green chroma), and b* (yellow-blue chroma) all decrease with increasing *ω*. Three key mechanisms underpin this phenomenon: Water-induced light absorption: Water exhibits a light absorption effect. As ω increases, soil water content rises, strengthening incident light absorption and reducing reflected light intensity, this reduction directly leads to decreased L*, a*, and b* values^39^. Altered light scattering behavior: With increasing ω, water gradually fills soil pores. By occupying interparticle voids, water partially replaces the diffuse reflection at the air-particle interface with specular reflection at the water-particle interface^40^, this replacement traps light that would otherwise escape via diffuse reflection in soil pores (due to specular reflection), further lowering overall reflected light intensity. Modified soil structure: At low *ω*, soil particles are bound by cementitious substances (e.g., clay minerals), as *ω* increases, excess water disrupts the original cementitious bonds between particles, forming a water film, the water film’s lubricating effect increases interparticle spacing and roughens particle surfaces^41^, further reducing soil reflectivity.

*Fe*_*d*_ directly governs the soil’s inherent color^26^, as illustrated in Fig. 3, soil becomes progressively redder with increasing *Fe*_*d*_, this is because *Fe*_*d*_ occurs in the soil as iron oxides and their hydrates—predominantly hematite (α-Fe₂O₃) in the granite residual soil of Guangxi, when hematite coats soil particles as a cementitious film or aggregates into nuclei, the soil presents a brownish-red or bright red hue^42^.

## Conclusions

Taking granite residual soil as the research object, this study aims to explore the intrinsic correlations between water content, free iron oxide content, chromaticity parameters, and shear strength indices, verify the feasibility of non-destructive and rapid estimation of shear strength via chromaticity parameters, and provide a new technical approach for the non-destructive assessment of mechanical properties of geomaterials. Based on experimental research and data analysis, the main conclusions are as follows:

1. At a constant water content (*ω*), cohesion (*c*) of granite residual soil increases with increasing free iron oxide content (*Fe*_*d*_), while internal friction angle (*φ*) exhibits no significant change. At a constant *Fe*_*d*_, *φ* decreases with increasing *ω*, following a first-order exponential curve; in contrast, *c* first increases and then decreases with increasing *ω*. The resulting curve exhibits a high fit to a quadratic function, with *c* reaching its maximum value at *ω*≈11%.
2. Lightness (L*), red-green chroma (a*), and yellow-blue chroma (b*) of granite residual soil all decrease with increasing *ω*, albeit at distinct decreasing rates. L* exhibits a significant decreasing rate at low *ω* and overall follows a first-order exponential curve; in contrast, a* and b* decrease linearly with increasing *ω*. This differs distinctly from the changes induced by *Fe*_*d*_ variations: as *Fe*_*d*_ increases, both a* and b* rise, while L* exhibits no clear trend. Among these responses, a* undergoes the most pronounced variation. This is attributed to the fact that free iron oxide in Guangxi’s granite residual soil predominantly forms hematite—accounting for the soil’s red hue.
3. From the test results and fitting equations, it is concluded that when only *ω* and *Fe*_*d*_ are considered, *φ* and *c* of granite residual soil can be derived from L*, a*, and b*. This demonstrates that the shear strength of granite residual soil can be rapidly estimated via chromaticity parameters.

In summary, the chromaticity parameter-shear strength correlation model established in this study breaks the limitation of traditional shear strength tests relying on time-consuming mechanical experiments, enabling non-destructive and rapid assessment of the mechanical properties of geomaterials. It not only reduces test costs but also improves the efficiency and safety of on-site engineering investigations, providing efficient technical support for engineering construction in granite residual soil areas. Regarding the transferability of the results, the proposed correlation is not entirely site-specific, with the core applicable prerequisites of similar mineral composition (hematite-dominant free iron oxide) and comparable climatic conditions. Subsequently, we will conduct validation tests in other granite residual soil areas meeting these criteria to further clarify the model’s applicable scope and promote the engineering application of this non-destructive assessment method.

## Data Availability

All data, models, and codes generated or used during the study appear in the published article.
